# "The Hospital" Nursing Mirror

**Published:** 1900-12-22

**Authors:** 


"The Hospital" Nursing Mirror.

Being the Nursing Section of "The Hospital."

[Contributions for this Section of "The Hospital" should be addressed to the Editor, "The Hospital" Nursing Mirror, 28 & 29 Southampton Street, Strand, London, W.C.]

### Notes on News from the Nursing World.

### NURSES AND THE WAR.

As the year draws to its close the interest in the forthcoming list of New Year's honours deepens. That it will contain the names of many who distinguished themselves on the field of battle in South Africa is certain, and nurses are wondering whether, in addition to the names of some of the brave men who fought for England, those of some of the brave women who saved lives for England at the risk of their own will be included in it. We are satisfied that however this may be, the Queen will take care that the work of the nurses does not pass unrecognised. It may be found difficult to single out any special nurses for honours, though we are sure that if this is done, there will be no display of jealousy. But the issue of a medal to all who have served to the satisfaction of the authorities would be the most acceptable mode of acknowledging the part which women have played in the war.

### COMFORTS FOR THE SOLDIERS IN THE HOSPITALS.

We have frequent communications from the military sisters in the hospitals in South Africa. They often point out the difficulty of getting little extra comforts like tobacco, pipes, cigars, for the soldiers in the hospitals, and express gratitude to the few who have sent them gifts of this kind. On hearing this, Messrs. R. and J. Hill, Limited, of Shoreditch, the well-known wholesale tobacconists, made up a packet of their specialities for the soldiers in No. 6 General Hospital, Johannesburg, which they have sent out to one of the military sisters. The gift is most opportune and generous, and we shall be very glad to furnish any other firm with the names and addresses of military sisters, who would be extremely grateful for similar gifts for the soldiers in the hospitals under their charge.

### NURSING THE DAUGHTER OF LORD ROBERTS.

We hear that Sister Skillman, of St. Bartholomew's Hospital, one of the Princess of Wales's nurses sent out to the front last January, was specially selected to nurse the Hon. Miss Roberts through her illness from typhoid, and that the patient has, happily, made a perfectly good recovery.

### A LETTER FROM LORD ROBERTS.

Miss L. A. Mont-Wilson, matron of Cardiff Infirmary, who left England last June for South Africa, and has been serving as a superintendent nurse of Mr. Langman's private hospital, has now resumed her duties. During the time she had charge of the nursing arrangements Lady Roberts and her daughter came to the hospital twice, and afterwards Lord Roberts, who seemed very pleased with all he saw. Subsequently he gave her, in the following letter, permission to return home in consequence of Mr. Langman's time expiring:—

"'Pretoria, Oct. 7, 1900.

"'Dear Nurse Wilson.—You and the four Cardiff nursing sisters can leave towards the end of the month and return home. Hoping you have had a pleasant time in South Africa, and do not regret having come out.—Yours very truly,

"'ROBERTS.'"

### NURSES AT THE FRONT.

The Croydon Corporation decided some time ago to place in the Municipal offices a brass panel upon which was to be engraved the names of the Croydon Volunteers and local men in the Imperial Yeomanry who had been on active service in the Transvaal war. At a meeting of the Council on Monday it was reported by the General Purposes Committee that this brass panel, four feet by three feet, was ready for erection at a cost of £52, the design having been approved by the Committee. Councillor Moss asked whether it would not be possible to put the names of Croydon nurses who had gone to the front also on this brass memorial. He thought these nurses were quite as worthy of the honour in their own department as were the soldiers. The chairman of the Committee could not agree to the suggestion, saying that the line must be drawn somewhere. Nothing, therefore, came of Councillor Moss' suggestion.

### COMING HOME.

Nursing Sisters Bond and Noble are on their way home from the front for two or three months' leave. Stationed in Natal before the war began, they have thus had a longer experience of active service in this campaign than any other nursing sister, with the exception of Superintending Sister Dowse who, with Sisters Bond and Noble, was in Ladysmith throughout the siege.

### PRIVATE NURSING IN SOUTH AFRICA.

In view of the many inquiries we are constantly receiving about private nursing in South Africa, we give in another column the opinions of two correspondents on the spot who are both nurses of experience. It will be seen that they greatly differ in their conclusions, and that even the Army sister who arrives at the conclusion that the nurse with good health and a small amount of capital would be afraid to start in South Africa does not agree with the very sanguine correspondent who write on the same subject in The Hospital Nursing Mirror a few weeks ago. On the other hand, a late lady superintendent draws a gloomy picture of the position, and, having described life in South Africa as most perplexing, full of hardships, and race hatred, stigmatises it as the "Land of Lies." She is probably either a pessimist or her experiences have been exceptionally unfortunate. But nurses who are fairly remunerated and comfortable at home will none the less do well to think twice, and make many careful inquiries before they decide to embark upon private nursing in South Africa.

### CHRISTMAS AT THE CANCER HOSPITAL.

Christmas celebrations at the Cancer Hospital, Fulham Road, begin before the dawn of Christmas by the singing of carols in each corridor by the nurses and servants. The American organ is brought out from the chapel to start them, and the doors of the wards are thrown open so that the patients may hear. At breakfast-time the Lady Superintendent goes through the wards and distributes letters, cards, and presents—of which, alas! there are not enough to go round. This year the number is disappointingly small, and the task of allotting the warm garments will be a difficult one. "It certainly seems a little hard," said the Lady Superintendent, "that we cannot give every patient a present, especially as the hospital is fairly full." The patients have a good dinner of roast turkey and plum pudding, and each may have two friends to tea, when oranges and bonbons are provided. Those who are well enough sit up to the tables in the wards. In the evening there is an impromptu concert, for which the Lady Superintendent is responsible. The wards and chapel are decorated, each ward being left to the taste and ingenuity of the sister in charge; the ornaments, both of evergreen and coloured paper, are plentiful, but they do not remain upon the walls and ceilings very long. If anyone has any warm garments or other gifts to dispose of they would be thankfully accepted by the Lady Superintendent of the Cancer Hospital; the present supply will only be sufficient for about one-quarter of the patients.

### CHRISTMAS IN THE GOOD OLD STYLE.

Christmas is kept very thoroughly at the Brompton Hospital for Consumption. About 5 o'clock in the morning the nurses go round singing carols; then there is an early celebration of Holy Communion in the chapel, which is prettily decorated. The patients, through the kindness of friends, have a Christmas dinner of roast Turkey, plum pudding, &c., and for this about nineteen turkeys are required; usually about ten are presented, and the hospital provides the remainder, so that no one may be disappointed. A friend also sends yearly, and has promised to do so this Christmas, a hamper of evergreens, to help in the decorations, which are somewhat elaborate, and quite in the good old style. After dinner the patients spend the rest of the day in quiet amusements, or, as our representative was informed, "mild jollifications." On Thursday, two days after Christmas Day, there will be a Christmas tree, managed by the Lady Superintendent and friends who give the tree and a good many of the presents; and someone personating Father Christmas, in scarlet gown and venerable beard, will climb the ladder and make an opening speech, after which the gifts will be distributed. Those patients who are not well enough to come down to the entertainment room have their presents carried up to them by the nurses; each one receives a useful gift of warm clothing, as well as something pretty. Christmas is a very bright time at Brompton; everyone concerned—matron, nursing staff, doctors, and servants—throw themselves into the spirit of it with great zeal and energy; and patients, when leaving have been known to hope they may be in the hospital again at Christmas time. On Saturday there is a concert, given by the medical officer and friends; the usual Tuesday entertainment is omitted on New Year's Day on account of the full programme of amusements during Christmas week. The recognition of two of the plates given with The Hospital last week has given great pleasure to the authorities at Brompton; the "delicate tracery" is the work of one of the nurses, and is her own idea; while the York Gallery is represented in the picture of the decorated arch.

### CHRISTMAS AT THE ORTHOPÆDIC HOSPITAL.

At the Orthopædic Hospital, Great Portland Street, each sister gets up an entertainment in her ward, and everyone has a present. "Truth" and other friends send toys for the children, but otherwise, the matron said, "we are not overdone with toys." The London Needlework Guild has again this year sent a bundle of useful clothing; this the matron will distribute to the most necessitous cases, not necessarily at Christmas, but throughout the year as need arises. The festivities began this week with an entertainment given by Mrs. Muirhead Little and her children and friends. On January 12th a Christmas tree will be provided by the Committee; this is a private present from themselves. There will be a tea beforehand for the patients, who will sing carols with the nurses help. Tea will be at five o'clock and the tree at six.

### CHRISTMAS AT THE BRISTOL GENERAL HOSPITAL.

On Christmas Day there will be a dinner for the patients—roast goose and turkey, and plum pudding. The men will be presented with one ounce of tobacco, the gift of Messrs. W. D. and O. Wills. The women will receive a box of chocolate, the gift of Mr. J. S. Fry. The nurses will sing carols in the wards during the day. In the evening the matron, Miss Morris, and residents are at home to the nurses from 8 p.m. to 11.30. On the 26th Madame Clara Butt will sing to the patients; on the 27th there will be a concert on the male floor; on the 28th a concert on the female floor given by the nurses; and the 31st is Christmas-tree day. A free tea will be given in each ward, and the honorary staff, the students and nurses will have tea with the patients. Each patient will be presented with a gift of warm clothing and a small present off the tree. On January 3rd there will be a dinner-party for the sisters; on the 7th an At Home for the nurses; and on the 8th a servants' party.

### QUIET NIGHTS.

A house for the night staff has just been completed and opened at the Cancer Hospital, Fulham Road. It is separate from the rest of the building, but reached by a covered passage. The night superintendent has charge of it, and each nurse has a nice cosy little room; these are on two floors. The passages are distempered by a new damp-proof process. The house is a great boon to the night nurses, as being only used by them it is perfectly quiet and undisturbed during the day.

### THE SLANDER ON A SHEFFIELD NURSE.

A cruel and cowardly attempt to take away the character of Miss Clarice Battye, a charge nurse at Firvale Workhouse Infirmary, Sheffield, has been triumphantly frustrated, and the author has not only been convicted at the West Riding Assizes of slander, but ordered to pay £100 damages and costs. The allegations of the defendant was the more difficult to meet because it was vague; but, in effect, the plaintiff was accused by Mr. Frank Shelten of misbehaving herself at a public ball with a medical man, when the electric light was turned off for a couple of minutes. The utter groundlessness of the charge was demonstrated both by the trustworthy witnesses called on behalf of the plaintiff, and also by the failure, or fear, of the defendant either to enter the box or to call anyone on his behalf. But it required some courage on the part of Miss Battye to face the possible ordeal of cross-examination, and she is warmly to be congratulated upon the manner in which she has confounded her traducer.

### QUEEN'S NURSES IN SCOTLAND.

The annual meeting of subscribers to the Scottish branch of Queen Victoria's Jubilee Institute for Nurses has just been held, and we are sorry to find that Dr. Bannerman had to state, in his speech, that the Institute is languishing for want of support. The record of work done for the year ending October 31st, 1900, is admirable. Thirty probationers entered the Training Home for six months' district training, and of these 13 had received their two years' general hospital training by arrangement with the Institute. Thirty-five nurses completed their full training as Queen's Nurses in Edinburgh, an increase of two on the previous year. The Scottish Council are now responsible for 52 nurses and probationers. During the year six new branches engaged nurses and were affiliated to the Institute, and there are 199 Queen's Nurses in Scotland working under 111 Associations. The new cases nursed were 4,024, an increase of 157; and the visits paid 92,788, an increase of 11,152. Although the expenditure exceeded the income by nearly £350, this is an improvement on the previous year; but so long as a deficiency has to be met out of capital it cannot be said that the Institute is adequately supported. We are rejoiced, however, to learn from Sheriff Guthrie, Q.C., that it is proposed to extend the system in parts of the country where, owing to the poverty of the people, they cannot be expected to support the nurses; and we are satisfied that the more its claims are pressed the more liberal will be the response of the public on the other side of the Tweed.

### THE NURSES' CLUB AT DUBLIN.

The inaugural meeting of the Nurses' Club at Dublin, about which particulars have already appeared in our columns, was held last week at the premises, 8 St. Stephen's Green. The rooms are handsomely furnished, and already nearly 400 members have been enrolled. Owing to the subscription being very small, however, it is desired that at least 250 more nurses should join during the coming year in order to meet the working expenses of the club. Many medical men attended the opening function, and Dr. Myles delivered an excellent address, in the course of which he paid a warm compliment to Miss Huxley, the president, of whom he said it was due to her in a higher degree perhaps than to anybody else in the nursing profession in Dublin that the present condition of nursing in Ireland had been brought about. He also alluded in terms of praise to the energy, capacity, and efficiency of the Dublin nurses engaged in the war—"the nurses who carried their lives in their hands, just as fearlessly as the soldiers did on Spion Kop." Dr. Myles, in conclusion, said that every medical man in Dublin wished the club most hearty success, and expressed the hope that nurses would in due time, instead of occupying rooms, possess a club-house of their own.

### WET FEET AND COLD DRAUGHTS.

The body known as Plymouth Corporation of the Poor have been discussing complaints by the nursing staff at the Workhouse Infirmary. Dr. Collins, the medical officer, reported that the nurses were "constantly catching colds from wet feet and cold draughts." He also stated that when they returned to the Workhouse with wet clothes there was no suitable accommodation for drying them. It being asserted by some of the Guardians that alterations were to be carried out at the Workhouse which would give the nurses greater accommodation, Mr. Ham declared that even then there would be "only bare accommodation," and pointed out that at present they have to cross wet yards to get from one ward to another and to the hospital. Mr. J. Brown also did not think that the nurses received the consideration they deserved, but a very unsympathetic resolution, vaguely intimating that steps are to be taken to make the nurses "more comfortable," was carried. Wet feet and cold draughts are, however, too serious to be treated as matters of indifference, and unless the nurses at Plymouth are made "more comfortable" in this respect very speedily, we advise them to renew their complaints. The Plymouth Guardians have, indeed, made one concession, and agreed to purchase six cloaks for sheltering the nurses whilst going from the hospitals to the wards in cold and stormy weather. But this, which was denounced by some of the Guardians as "an unnecessary luxury," will only be a small alleviation.

### TO PENSION FUND NURSES.

We are asked by the Hon. Secretary of the Junius S. Morgan Benevolent Fund to remind members of the Royal National Pension Fund that if they wish their annual contribution of 1s. to their Benevolent Fund to be included in this year's collection, they must kindly send it to The Secretary of the Pension Fund, 28 Finsbury Pavement, before December 29th.

### EXAMINATION AT LEWISHAM INFIRMARY.

The examination at the end of the first year and the completion of three years' training was held last week at Lewisham Infirmary. In the case of the probationers the examination by the medical superintendent lasted two hours, six questions were asked, and four attempts to answer were allowed, three being compulsory. The third-year nurses had two groups of six questions given them by Dr. J. Rose Bradford and the medical superintendent, the time given was three hours, and three attempts to answer three questions in each group were permitted.

### INFIRMARY NURSES AND MASSAGE.

Four of the sisters at the Infirmary, Isleworth, are among those who passed successfully and gained certificates at the examination held by the Incorporated Society of Trained Masseuses on November 27th and 28th, namely, Miss M. Parker, Miss K. Smith, Miss H. Raggett, and Miss M. Brooks. The Brentford Board of Guardians, on the advice of the medical superintendent, arranged with Miss Knight, of the Society of Trained Masseuses, to give a course of lectures and demonstrations at the Infirmary, where massage is used in the treatment of many of the cases.

### SHORT ITEMS.

The Godalming Nurses' Home will benefit to the extent of nearly £30 from the *Tableaux Vivants* and concert given on behalf of that institution last week.—The Christmas decorations will be very simple at the London Throat Hospital, Great Portland Street: most of the patients will go home, and those who remain will be cases of such serious nature as to make it undesirable for much to be done in the way of celebrating the festive season.—The annual bazaar held by the Ladies' Committee in aid of the patients of the Orthopœdic Hospital, Great Portland Street, this year brought in over £220.—At Reading, on Tuesday, Miss T. M. Masters, a nurse, was awarded by a special jury £225 damages in a breach of promise action, remitted from the High Court, against Mr. J. R. Chambers, a dentist, of Oxford and Reading. At the defendant's request Miss Masters had given up following her profession. —Miss Ellen Langstaffe, the new matron of Victoria Hospital, Worksop, was sister in charge of the Borough Sanatorium, Eastbourne, not East Ham.

### Lectures on Nursing for Probationers.

By E. MacDowel Cosgrave, M.D., Lecturer to the Dublin Metropolitan Technical School for Nurses.

### DIET FOR THE SICK.

(*Continued from page* 126.)

In acute cases where there is failure of digestion and absorption, the diet should be chiefly if not altogether fluid, and composed of milk, possibly diluted, broth, barley-water, &c. As convalescence progresses so the diet should be increased. First a beaten-up egg, a little arrowroot, or a little biscuit or bread and milk may be added. Then a little bread and butter and a light pudding. Then whiting, sole, plaice, or trotters. Then a little chicken. Then a chop.

Some diseases require special diets:—

In Bright's disease the kidneys are failing, and so they should be given as little as possible to do; so whey, carbohydrates, and a minimum of proteids is the best diet. In diabetes the liver is failing to store up sugar which escapes in great quantities into the blood and is given off by the kidneys. So sugar and starch must be avoided, and proteids, gluten bread, almond rusks, &c., are given. In gout, much meat or broths is injurious. In rheumatism, milk should be used sparingly.

### XXI.—THE HYGIENE OF THE SICK-ROOM.

The wards of hospitals are specially built for the treatment of sickness, and the furniture, &c., are appropriate, so keeping things in order and attending to ventilation, cleanliness, and so forth, are the chief requirements.

In private nursing it is different; there is a choice of rooms, a choice of position for the bed, a choice of furniture, and many other choices, so it is well to know the chief hygienic requirements, and to aim at securing as many as possible. The better the conditions of the sick-room, the better the chances of recovery, and the quicker convalescence.

The sick-room should be large and lofty in order that ventilation without draught may be easy. It should be light; as surely as a person is kept from light, so surely will the red corpuscles fail, and anæmia result; in health deprivation of light cases a person to become pale, listless, and out-of-sorts, and such a tendency must be unfavourable in illness. The best aspects range from south to west. A north or east aspect is bad; early morning light is not of much importance to invalids, and may even be an injury, as some cannot sleep when a room is light, so such an aspect either lessens their hours of sleep or leads to the windows being shuttered and curtained to keep out the light, with the result that ventilation is impeded.

At the end of the day it is just the reverse; the afternoon often tires invalids, and with the fading light chilliness comes on. If now the blinds are drawn up, the bright gleam warms and cheers the patient. The sick-room must have an open chimney, and except in very warm weather a small fire will be found to assist ventilation without unduly heating the room. The window should open both top and bottom, and the air of the room should never be allowed to become stuffy; the best test for stuffiness is to go into the sick-room directly after a walk in the open air. The sick-room should be thoroughly cleaned, if possible, before the invalid enters into possession. All useless furniture should be removed, especially wardrobes and chests of drawers containing clothes; all woollen curtains and large carpets should also be removed; the walls should be dusted, the floor and woodwork wiped with a cloth wrung out of hot Condy's fluid and water.

Then the necessary furniture should be selected, such as the bed, a small table to stand next the bed, another larger one somewhere else, and yet another outside the door, all of which should be covered with clean napkins. A strip of carpet may be placed beside the bed, another may run to the door, and another, or a rug, may be placed before the fire-place. A few fresh flowers may be placed in a glass where the patient can see them, but they should be put out of the room at night-time, and next morning all fading blossoms should be removed, and the end stalks of the remainder should be cut off, and they should be placed in fresh water. The bedstead should be of iron, the best shape is narrow and long, and there should be a wire mattress and a good firm hair mattress. It should be placed in an airy part of the room, if possible not facing the light, and not against the wall, as it is hard to nurse a case properly if both sides of the bed are not easily reached. Sometimes, as in great weakness, when a patient has often to be raised on his pillow, it is a good plan to keep the head of the bed out from the wall. If the bed has to face the light, a screen should be sometimes placed to prevent the glare falling on the patient's eyes, yet keeping the room bright. It is a good plan to have a second bed, to which the patient can be moved whilst his own is being thoroughly aired and freshly made.

The temperature of the sick-room will vary with the time of year—probably in winter it will be sufficient if the temperature does not fall below 55°, but at night even this will necessitate careful attention to the fire. In summer 60° may be adopted; in croup and bronchitis, and in the case of old people, the temperature may be raised to 65°. The temperature should be read from a thermometer hanging close to the head of the bed; if, as is often done, the thermometer is placed over the mantelpiece, or on the wall close to the chimney, it will mark too high, as what is wanted is to know the temperature the patient is experiencing.

A small screen should be provided so as to be able when necessary to keep the glare of the fire off the patient, or to cut off some of its direct heat. Small briquettes and compressed peat make a capital fire—there is not much flame, there is a clean smell, and it will smoulder on for a long time. The best poker is a piece of wood, and an old glove should be kept so that fuel may be noiselessly added.

Although the arrangement of the system of drainage is out of the nurse's control, there are some points to which she should direct her attention. Any smell from bath or lavatory may mean untrapped pipes (that is, pipes opening into the sewers without being cut off by a portion kept filled with water, so that sewer gas may not be able to push by). This should be reported, as it may prevent or delay recovery.

Drinking-water should never be drawn from a cistern, as cistern-water is always liable to be contaminated by sewer and other injurious gases. If there is even a suspicion of this every drop of water used in the sick-room should be boiled, and afterwards aerated by being poured backwards and forwards from jug to jug. Special attention should be paid to the closet, especially if near the sick-room; the door should be kept closed, the window open, and free flushings with creolin solution or other disinfectant should be used, especially after motions are emptied down. If the drains are foul chloride of lime is best, as it cuts away deposit. Motions should be received in a vessel containing creolin solution, and at once removed from the sick-room and emptied, unless they are to be kept for the doctor's inspection, when they should be placed in the closet and covered with a cloth wrung out of creolin solution. The same pre caution should be taken with vomited matters.

The cleanliness of the patient must be carefully attended to, as free action of the skin is of great importance. The hands and face should be frequently washed, and the skin sponged with soap and water, at least once in the twenty-four hours, due care being taken not to uncover too much at a time, and to avoid chill; a little ammonia added to the water is refreshing. The teeth should be cleaned with a little cotton wrapped round the end of a piece of stick; dilute glycerine and boracic acid makes a nice mouth-wash.

(*To be continued.*)

### The Nurses of the London Temperance Hospital.

### A CHAT WITH THE MATRON.

By Our Commissioner.

It is the rule at most hospitals where paying probationers are received to limit the number; but at the London Temperance Hospital none but paying probationers are received. When I saw the matron, Miss Lucas, in her pleasant room, at the admirably arranged building in Hampstead Road, I asked her how the system works.

"We never," she said, "have any difficulty in obtaining probationers. People tell me they cannot understand how we get them. Perhaps the fact that everything is so comfortable here may account for it."

### Terms of Payment.

"What are the terms of admission?"

"Candidates who desire to enter for three years are required to pay a fee of thirty guineas, but in the second year the probationers receive a salary of £15, and in the third £20. We admit one, or two at the most, for a period of one year, and they pay a fee of a guinea a week."

"Can those who come for one year only stay on?"

"Yes, and as a matter of fact many of them do so. I always explain to those who enter for twelve months that the one year's certificate is of no professional value. Still, there are girls who come for a year and have no idea of taking up nursing as a profession."

"You say that you only admit two at the most each year. What is the objection to receiving more?"

"The only objection is that our accommodation does not admit of it. Otherwise, I should not mind taking more, though it would mean additional teaching for the sisters. All our probationers belong to the same class, and, I think, get on very well together.

### A Prospective Private Staff.

"Has the question of four years' training been raised here?"

"Never. But our nurses very frequently want to remain at the end of their three years' engagement. We are often able to keep them on the staff, and I hope we shall be in a position to do so more generally when we have a nursing home?"

"How soon do you expect to get one?"

"It is probable that we may be able to start it next year. At present, the house bought by Mr. George Cadbury for us about three years ago is used for the nurses of the hospital. We hope to have another for the private nursing home, although we have not collected any funds."

"How many nurses do you expect to have attached to the private staff?"

"About nine. We have constant inquiries for private nurses. Of course, none would be sent out till they had completed their three years' training at the hospital. The private staff would be quite distinct from the hospital staff, but under the control of the matron. If we had one of the private nurses in the hospital we should pay for her services."

"What accommodation is there for nurses in existing circumstances outside the hospital?"

"The house, which is connected by a dining-room, built by Mr. Cadbury, with the hospital, contains a sitting-room and six bedrooms. The proposed home is next to it, and we should be able to make a similar connection with it."

### The Hospital Staff.

"I suppose your staff is not a very large one?"

"There are thirty-five sisters and nurses—a very good staff, I think, for 100 beds. But we should like more in order to increase the amount of off duty. There are four ward sisters, a night sister, a casualty sister, and a home sister. The casualty and the night sister are additions this year. There had previously been no night sister in the hospital, but the work has grown considerably, and I have found a night sister to be of great help."

"What are the hours off duty?"

"The day nurses are off duty from 11 a.m. to 1 p.m., or 2 to 4.30 p.m., or 6.30 to 8.30 p.m. They have one day every month, viz., from 9 o'clock one night to 10 o'clock the next. On Sundays they have from 2 to 10 p.m., or 10 a.m. to 1 p.m., or 2 to 4.30 and 6 to 10 p.m,, or 10 a.m. to 1 p.m. The night nurses are off duty from 9.30 a.m. to 12.30 p.m., or 5.30 p.m. to 8.30 p,m., and one night off every month, viz., from 1 o'clock one day until 5 o'clock the following. I confess, as I suggested before, I should like them to have more time off."

"What is your ideal time of off-duty?"

"Four hours a day. Still we do not have such long hours on duty as in many other hospitals, and none of the nurses go on duty till eight in the morning. As to holidays, the probationers have three weeks and the staff nurses and sisters a month."

### The Instruction.

"You might tell me about the course of instruction."

"Each year courses of lectures are given to the nurses and probationers by the visiting staff. The examination is completed at the end of the second year; afterwards, in the third year, the nurses can have dispensing lessons for six months. These lessons are given three times a week, and prove very useful. Dr. Collins usually lectures between October and Christmas, and Dr. Fenwick the next three months. The first year probationers have to pass an examination on my lectures. This year I am dealing with the Theory and Practice of Nursing."

"What happens if they fail to pass an examination?"

"They have to try again. They must make half marks.

### The Temperance Question.

"How does the fact that this is a temperance hospital affect the nurses?"

"The nurses while they are in residence are required, in common with all the staff, to be total abstainers. All the nursing is done on the same principle, although we administer alcohol occasionally, but never in the form of brandy or whisky."

"How about diet?"

"It is on a very liberal scale. There is no difference in the quality of the food used from the doctors down to the servants, and we try to get as varied a diet as possible. I think the best proof of its satisfactory character is that I have never had a complaint about it. Either the matron or the home sister presides at the meals, and we see that everything is right. The night sister looks after the night meals."

"Is there any difference between the arrangements for the meals of the staff nurses and sisters?"

"Only that the sisters dine with the matron in the evening, while the nurses have supper."

### Sleeping Accommodation.

"Do the nurses have separate bedrooms?"

"With two exceptions, and these are usually two friends who like to be with each other, or the last two probationers. Until we opened the new ward last year, each nurse had a room to herself."

"How are the wards staffed!"

"According to the size, which varies. One of sixteen beds has in the day a sister, a staff nurse, and two probationers; and in the night a nurse is in charge, with a probationer between two wards. The night sister has six nurses and three probationers."

"You have no ground attached to the hospital?"

"No; our garden is on the roof, and it is rather smutty. The nurses have a comfortable sitting-room, but I like them to go out every day if the weather is at all fit. We have a number of enthusiastic cyclists, and being so close to Regent's Park is an advantage. Occasionally, tickets are sent for the theatre, and I am always glad for the nurses to have amusement and change of scene. They work all the better for it."

"We were exceptionally busy," continued the matron, "in the usual summer holidays this year, owing, perhaps, to University College Hospital being closed; we had to put on an extra nurse in August and September. There were 1,200 patients treated in a week in the casualty ward, apart from the out-patients."

Nurses in South Africa.

"Have many of your nurses gone to South Africa?"

"Three. One sister has just come back. She went to Bloemfontein in March last, and, contracting typhoid, was invalided home in August. She has been here nine years, and is glad to be with us again, though she values the experience she has had of nursing the sick and wounded."

"You succeeded Miss Orme, I think?"

"Yes; I am the second matron. The hospital is twenty-seven years old, and Miss Orme, who worked very hard in its interests, was here for twenty-five years. I was trained at the General Infirmary, Leeds, and was sister at Radcliffe Infirmary, Oxford, for five years."

Subsequently, the matron showed me over the hospital, and the house in which some of the nurses reside. So far as space permits, the provision for the latter is all that could be desired.

### Our Christmas Distribution.

We wish to thank our readers most heartily for the pleasure they have afforded us this year by their generous response to our appeal for the sick poor when leaving the hospitals. The gifts are all the more valuable in our eyes, as they do not come from rich and leisured folk, but, for the most part, from busy nurses who have already great calls upon their time, attention, and money. The numerous parcels we opened testified to the thoughtful kindness of the donors, for every imaginable warm and useful garment was found inside them.

Two kind nurses sent a number of warm and pretty outdoor hoods to shield the heads of the babies and little ones of all ages. Some of the smallest of these we sent to the East End Mothers' Home, whence several little strangers will emerge warm and protected, and not dependent upon a little bit of an old shawl, easily displaced, which is so often the case. Here, too, we sent two tiny cotton garments of most dainty though durable description, besides numerous cosy woollen articles of clothing for the babies, and nice, warm petticoats, vests, and shawls for the mothers.

We had an unusually large number of presents for the children, and thus were able to send parcels as well to the East London Hospital for Children, the North-Eastern Hospital for Children, The Paddington Green Children's Hospital, The Plaistow Maternity Charity, the Hospital for Women and Children, Waterloo Road, the Children's Convalescent Home, Great Yarmouth, the St. Vincent's Hospital, Gort, Ireland, and to four general hospitals, Charing Cross, King's College, the Metropolitan, and the Westminster Hospitals. To these latter we apportioned only clothing for men and women, and were glad to have so many nicely knitted socks to add to each parcel. The beautiful flannel petticoats, warm vests, bodices, shirts, and stockings, will help to send out many patients well equipped to face the winter weather.

It is always our custom to add our own mite to the fund, and this year we expended it principally in warm under-garments for men, as our readers send us fewer of this description of article. Some friends were kind enough to send us some toys and scrap-books, and we have to thank Messrs. Garrould, of Edgware Road, for a nice present of toys for Christmas trees. Some of these we added to our parcels of clothing, but the majority we sent to the Victoria Hospital for Children, Chelsea, as we had no garments for them this year.

It is a difficult as well as a pleasant task to apportion such a varied assortment of articles, and to select the recipients. We try to distribute where we think the gifts will be most needed, and to send samples of each description of garment in every parcel. But of course of some we have many more than of others. We welcomed the much larger proportion of warm nightgowns, petticoats, and little dresses for children on this occasion, and congratulate our readers on the neat handiwork which was conspicuous generally. In all we received 214 garments, which encourages us to hope that next year our collection will be larger still. We shall be equal to the task of distributing any number.

In addition to the contributions already announced we have to acknowledge the receipt of parcels from the following readers:—Mrs. Roberson, Baltic Villa, Henley Road, New Barnet; Mrs. Morris and Nurse Knight; Miss M. A. Stoutt, Mill Road Infirmary, Liverpool; "M. R.," Ryde; Nurse Lucy; Miss Dawson, Harlesden; Miss Blackett, 9 St. James' Street, Winchester; Miss F. Burdett, Mancettei, St. Michael's Road, West Bournemouth; Policy-holder No. 1,081; Miss Edith Vecoers, Fulwood, Preston; Miss Bedford, 13 West Cliff Road, Ramsgate; Nurse Oxley, Chevin View, Rawdon, Leeds; Nurse Clarke.

### Lady Eden on Nursing and Nurses.

A Christmas fair and sale was opened last Friday on behalf of the Gainford District Nursing Association, Darlington, by Lady Eden. She observed: "I could not resist the request to come and speak on behalf of nursing, which has so great an interest to me. I believe that we have all vocations in life; I mean the instincts with which we are born predominate in our characters and our lives; and I have always felt that had I not married and had a family to fuss and worry about I should have become a nurse. It is a real satisfaction to know that nursing institutions are increasing throughout the country, and it is now almost difficult to realise that only a generation ago there was no English training school for nurses in existence. Their multiplication and development have been so rapid that the comparative novelty of the movement has been overlooked. The history of nursing was really very interesting, although we cannot trace it back to the first sick person in the world. I wish we could, for it would be interesting to know how his neighbours treated him. I believe that many centuries before the Christian era 'houses for the sick' were maintained by Hindus and Buddhists, but there is no evidence of their having had nurses in their establishments. It was in the fourth century that the first demand for hospitals was made, and the work then seemed to have been principally done by religious societies." In conclusion Lady Eden said: "Now there is not a town and hardly a village that does not have its district nurse. Now, too, I think it is felt that a district nurse must have the very best training which can be obtained—and quite right too, for she has to deal with all kinds of emergencies, and often has to carry out the doctor's treatment when he is miles away, so that the old idea that 'anybody' would do for a district nurse has quite died away. It has always seemed to me that of all employments open to gentlewomen there is none more suitable than nursing, and especially nursing among the sick poor in their own homes."

### Private Nursing in South Africa.

By an Army Sister.

One so often hears the question, "Is there a good opening for trained nurses in South Africa?" Provided that nurses are willing to adapt themselves to colonial life and customs, and also that they are in possession of some means to enable them to wait until they become known to the medical men, I should reply in the affirmative. The majority of nurses work on their own account, although there are in the larger towns nurses' institutes worked on the same lines as those at home, where certificated nurses of three years' training are taken on, assuming, of course, that their personal character is satisfactory. It is useful for a nurse in this country to possess a certificate of maternity training, especially if she intends taking up work in a country district of the colony or up country. In Cape Town there has been a great demand for nurses lately, but this is mainly owing to the war, many of the nurses who usually take private cases having been employed on transports for the care of the convalescent soldiers invalided home; and, again, temporary nurses were engaged at the general hospital, where the staff was found inadequate owing to the great number of typhoid cases. Of course this is an abnormal state of things; still, there is every prospect of a thoroughly trained nurse doing well if she is not afraid of work, for private nursing has some drawbacks here, even more than in England.

### Fees and Cost of Living.

A nurse has often to wait upon herself, which, of course, no sensible woman would object to; but it is trying sometimes, especially when one has charge of an anxious case. It is, however, unavoidable, as wages are high and fewer servants are kept by middle-class people than at home. The fees are much the same, £2 2s. for medical and surgical cases, and £3 3s. for infectious cases. Rooms are more expensive, and in boarding-houses the terms are from £6 per month. Washing is done at the rate of 10s. a head per month. Money does not therefore go very far between cases. In Cape Town most of the surgical cases are treated at a private surgical home in the suburbs, or in another private hospital where any medical man may send and subsequently attend his own cases; and there are also private wards in the general hospital.

### Prospects in the Centres.

One can say little about prospects of nursing in Johannesburg. Until the war, it had been for some years "the happy hunting ground" of nurses. The fees are higher than in other towns, simply because living is more expensive, and all salaries are high. When peace is declared, and the refugees return, there will be a splendid opening for nurses. The climate is excellent, and although the cost of travelling from the Colony is great (£12 single fare), a reduction is made for nurses in uniform. In Bloemfontein also there are some openings for trained nurses. Although there are nurses there at present, there is room for others, and the climate, to one who can stand a fair amount of heat, is an attraction. There is always a great amount of typhoid in the summer, owing, according to some authorities, to the dust; but I should not like to vouch for the accuracy of this statement. Kimberley, again, presents many attractions, and in Rhodesia there are openings, and there will be more when the country is developed. In Buluwayo, the principal town, there are some private nurses, but there is room for others; there are also openings in some of the smaller towns of the Colony, although I fear that there is scarcely sufficient work to keep a nurse constantly employed. Still, living is cheaper than in the large towns, and country life has many fascinations.

### No Fortunes to be Made.

I have only briefly touched upon a nurse's prospects of private work in this country; for those who prefer hospital appointments I will give a few hints in a subsequent paper; but any nurses who come out with an idea of making a fortune will find that there is little more chance of doing so than at home in our profession. Nevertheless, with good health and a small amount of capital, no nurse need be afraid to start for South Africa. One meets with much kindness and plenty of consideration and courtesy from the medical men, who, being frequently "Guy's" or "London" men themselves, give a kindly welcome to an English nurse, and are ready, if she prove herself worthy, to give her a helping hand.

---

### THE OTHER SIDE.

By a late Lady Superintendent.

Perhaps a few words from one on the spot who is going, and has gone, through a fiery furnace, may help to deter not only nurses, but other workers also, from rushing out to South Africa without studying the situation, asking a good many questions, and looking before they leap. The woman who has leisure to read the newspapers will have discovered for herself that there is something wrong in this colony, radically wrong, politically and socially wrong, and before this can be righted workers will do well to struggle on in the old land instead of rushing out here at their own expense and risk, only to be treated with contempt and callousness; possibly lose health, strength, and hope, and then be left to starve.

### A "Land of Lies."

Life here is most perplexing, full of hardships, race hatred, and verily the "Land of Lies." A small salary is always given for very hard work, and although £60 a year may look very liberal terms in black and white, and a plausible letter behind it, one may soon discover by judicious questioning that one is far better off with £25 a year in England and the other colonies than that sum here. I would advise nurses, to fight shy of nursing homes, particularly if they are gentlewomen and well trained in their profession. For middle-class women who are very healthy, young, and not over-refined, there may be a field of labour if they understand anything about midwifery and monthly cases, but even those will soon discover that they must compete with coloured and unskilled workers, do much menial work, spend all their earnings, as everything is ruinously expensive, and finally have the mortification of being boycotted and disliked if they decline to undertake medical work between their cases.

### No Social Position.

Nurses, too, here have no social position as a rule, they are neither valued nor wanted after their work is done. Excepting in very isolated cases they are badly fed, housed, and treated with distrust and suspicion. Utterly regardless of the fact that a nurse is human, she is expected to sleep anywhere, go anywhere, do anything, go without fresh air and sleep, submit cheerfully to the filth, dirt, rough and tumble of an ordinary South African house, and then hear afterwards evil things said about her; for in this land there is no loyalty, the labourer is not worthy of her hire. She cannot believe anyone, no one either believes in her or cares to know her unless she is deceitful and insincere, and when settling day comes there is usually some quibble about fees or washing, &c., which is very trying to over-taxed body and mind, and sometimes there is no money to pay her.

### Fairy Tales.

It is not my intention to speak of my own experiences, and the way I have been treated here, but bitterly do I regret that I ever put my foot upon South African soil. The breakdown among nurses is very bad. The climate is certainly trying although not unhealthy, but the saddest part of all is that good, honest conscientious work is not appreciated as it is in the other colonies Everyone muddles through somehow, and if one objects to this or expresses surprise and desires something better, one is met with dislikes and prejudices, and many reforms are needed, and many lessons will have to be learnt before good can be done or come out of South Africa.

### Christmas Books.

### BOOKS FOR EVERYBODY.

### MARCHESTER STORIES.

(S. W. Partridge.)

THE "Marchester Stories" are a collection of pleasant, wholesome tales, by the Rev. Charles Herbert. Although the people and scenes belong to Marchester, there is plenty of variety in the sketches, so that everyone may find something within the book to suit his or her taste.

### A TWENTIETH-CENTURY PARSON.

(Skeffington and Son.)

IT is very quaint when reading of a personage in the twentieth century to do so in pages written from a standpoint which accords with the sentiments which animated writers of the earlier years of the nineteenth century. The parson, who is the person in question, is pleasant, manly, simple, and perfectly human. He finds himself in the uncongenial atmosphere of narrow-mindedness and prejudice. We are glad when he overcomes the obstacles which assail him. We have not so much sympathy with the young gentlemen and ladies who enter into the story. Mr. Sugden has not managed to garb them in a fashion familiar to this generation, and we find oursselves out of touch with their manner of conversation. But the book will fulfil a difficult want to supply: it will serve as an excellent present to elderly persons who live a secluded life.

### AS A WATCH IN THE NIGHT.

(Chatto and Windus.)

THIS last book of Mrs. Campbell Praed is what one might call ultra-modern in its attitude towards life. The story is called a drama of waking and dream in five acts. The first modern symptom is the transference of the veil of romance from the "young girl" to the woman of mature age. Then the doctrine of predestination, or irresponsibility of the individual, is most prominent, and the mysticism of Swedenborgianism is woven in the drama throughout. Dorothea Queste in her previous life was a Roman lady who murders her husband. She enacts the main features of her life over again as a modern Englishwoman, but through suffering and renunciation she makes to atonement. The book is bewildering, but it is full of emotion and dramatic interest; it will shock many and mystify not a few readers.

### THE WORLD'S GREAT SNARE.

(Ward, Lock and Co.)

"THE World's Great Snare," is the title of a book in which the search for gold, the record of evil passions, the misfortunes of the innocent, the relation of many dangers and difficulties are to be found. The work is by E. Phillips Oppenheim. Its claim to popularity is based upon the chain of stirring incident which is crowded in to it.

### THE BAG OF DIAMONDS AND THREE BITS OF PASTE.

THIS title covers four stories, the first being the bag of diamonds and the others the paste. We are unable to distinguish the respective values. All four stories are full of exciting incidents. In the first a bag of diamonds excites the cupidity of some adventurers, and much crime and misfortune result. In the second still more fabulous wealth leads to murder and general unpleasantness. The third shows how exceedingly unreasonable and objectionable 300 female emigrants can make themselves to the officers and crew of the ship in which they are sailing. The officers are models of self-control and good sense, and ultimately they triumph over the turbulent elements, both female and marine, and after shipwreck and disaster return to port safely and happily. An amicable settlement between the best behaved of the girls and the estimable officers is hinted at as a conclusion. The last story of the series is concerned with jealousy and attempted murder. But in common with the other stories in the book the author (Mr. George Manville Fenn) arranges a happy ending.

### BOOKS FOR BOYS.

### RED, WHITE, AND GREEN.

(T. Nelson and Sons.)

THE times of this story are placed, to use the words of the book, when "Louis Philippe, King of the French, had been driven into exile; Sicily had revolted against King Bomba; insurrections had arisen in Madrid; the whole of Germany had been, and was, in a state of turmoil; the Prussians had conquered Poland afresh." The story opens in Vienna. The city is in a state of insurrection; part of the army has joined to overthrow the Government. Hungary is struggling for its independence, and the narrative is told in the words of a young Hungarian, who, with his brother, joins in the struggle. Then come the stirring scenes of many battles, defeat, captivity, and finally the execution morning. Mr. Herbert Hayens keeps up the interest all through, and the historic episodes of his tale are little known to most English readers.

### THE MANDARIN'S KITE.

(Skeffington & Sons.)

THIS title covers the series of remarkable adventures which befell little Tsu-foo, the small son of a mandarin, and Cyril Deane, an English boy. By means of the mandarin's kite they accidentally visit the planets occupied by the signs of the zodiac, which are connected by an aërial railway. They returned to earth once more in an air-ship. The illustration in which Tsu-foo and Cyril are represented greeting Leo the Lion will give an idea of the clever and appropriate drawings which are scattered through the pages of the book. The work is by Mr. G. E. Farrow, author of the "Little Panjandrum's Dodo." Those who are acquainted with this amusing narrative will recognise the style of authorship. To others we would say that it mainly consists of dialogue full of the bright and clever nonsense of the type to be found in "Alice in Wonderland." It is a capital book for the young ones, and will amuse their elders by the way.

### KHAKI IN SOUTH AFRICA.

(Messrs. George Newnes, Limited.)

"KHAKI IN SOUTH AFRICA" is a handsome album illustrating the war in South Africa, with brief descriptive letterpress. The pictures number no less than 140, all true to life, as they are mainly reproduced from photographs. The scenes are most graphic, and form a complete education in the most varied military operations and scenes in which our troops took part. The many portraits are admirable. The book has a fine scarlet binding, and is thoroughly well got up.

### THE CAPTAIN.

(Messrs. George Newnes, Limited.)

A VOLUME of "The Captain," that excellent magazine for boys, affords an excellent Christmas present. It is a young competitor in the field of serial literature, and is bright and up to date. It contains just what our boys like, and much instruction pleasantly imparted. The binding is very attractive, whilst paper, type, and illustrations are all excellent.

### THE ROMANCE OF THE SOUTH POLE.

(Thomas Nelson and Sons.)

MR. G. BARNETT SMITH gives in one volume the history of those thrilling adventures which have attended the expeditions into the regions of the South Pole. This is a subject which never palls, and the idea of giving an account of all the expeditions under one cover is a good one. The death of Captain Cook forms the subject of one of the illustrations, which add to the interest of the book.

UP THE CREEKS.

(Thomas Nelson & Sons.)

THIS is a capital little story, simply told by Mr. Edward Shirley. There is not a word too much, and yet the exciting situations in which Dick Trentham, finds himself in West Africa are brought vividly before us. After being besieged by the Chief London Pride in a wooden hut, and after a desperate resistance he is captured with his faithful attendant, the Kroo boy "Mary Ann." He is eventually rescued in the nick of time, and all ends happily. The little volume would make an admirable and inexpensive addition to the Christmas stocking of any schoolboy, who will delight in its pages.

BOOKS FOR CHILDREN.

GRIMM'S FAIRY TALES. (Ward, Lock and Co.)

WHAT more delightful present for either small boys or girls could be found than "Grimm's Fairy Tales." Messrs. Ward, Lock and Co. have brought out a complete and new edition of the collection this Christmas. The book has an attractive scarlet binding, it is well and clearly printed on good paper, and has excellent illustrations. The tales themselves are too well-known to need comment.

THE MAGAZINES.

THE volume of the *Sunday Magazine* contains a mine of interesting literature for wet Sundays at home. There are interesting articles on religious and secular subjects, tales, verses, and illustrations. The type is legible, and the book handsomely bound. The volume for 1900 is ready in its handsome binding.

*Good Words* maintains its character for sound and interesting literature. The bound volume for 1900 is a gift which will find a welcome everywhere. The motto "Good words are worth much and cost little" is quite true in this case. There are numerous serial and short stories, biographical and science papers, notes on travel, literature and miscellaneous subjects, and excellent illustrations.

THE Christmas number of *Cassell's Magazine* contains a surprising amount of literature and some beautiful illustrations. Within the 160 pages of which it consists are to be found eleven stories, poems, and articles on various interesting subjects, amongst which is one on the Prince of Wales's horses, illustrated, ice-boats and boating, also illustrated, and many others. *The Leisure Hour* shows its seasonable character by an attractive picture on the cover. Within it contains a special story by Ethel Turner, short stories for Christmas, and numerous articles to please all tastes. *The Windsor Magazine* devotes a number of its pages to the London Hospital. Numerous illustrations show where the work is done and by whom. Mr. Mackenzie has collected a number of interesting facts about the hospital which we hope will incite the readers of the *Windsor Magazine* to remember the sick folk in Whitechapel at Christmastime. There are the usual interesting stories, other literary matter, and illustrations in the number, besides which a novel by J. C. Snaith, entitled "Mistress Dorothy Marvin," is issued gratuitously as a supplement.

### Novelties for Nurses.

WOOLLEN UNDERWEAR.

By OUR SHOPPING CORRESPONDENT.

THE system of woollen underwear as introduced by Dr. Jaeger has advantages that require only to be known to be widely appreciated. For invalids and others exposed to chills the benefits to be derived from clothing made entirely on this principle is simply invaluable. A visit to one of the numerous "Jaeger" depôts at, say, 126 Regent Street or 456 Strand, Charing Cross, would be sufficient to convince the most sceptical of its superlative advantages over every other system of dress. Though the natural colour of the wool is adhered to in most cases, yet the same results can be secured in white, pink, or blue, according to the taste of the purchaser. I particularly admired some really delightful-looking nightgowns made in white taffetas, a material as soft and fine as silk, trimmed with delicate torchon lace and richly embroidered, for 25s., and were of opinion that the most critical lady could wish for nothing more dainty or becoming. There were also petticoats, vests, and other articles equally effective, while for real comfort there was nothing that took my fancy more than a bed-wrap of the "Nightingale" shape, made of thick, fleecy, natural coloured woollen material, which was as warm as swan'sdown and as light as a feather. This for an invalid would be a never-ending comfort, while for those who preferred it there are dressing jackets with wide roomy sleeves to slip on easily over the nightdress. One of the great specialities, however, appears to be the varied and really excellent assortment of belts, obstetric binders, and corsets made on strict anatomical principles, and when so desired to ladies' own measure. On page 7 of the catalogue will be found an illustration of the "Pregnancy" belt, an ingenious arrangement whereby all weight is taken off the abdomen and pressure relieved. It also relieves pain in the back and is of great assistance in walking. Another variety is one specially recommended for corpulency: it is provided with a non-yielding front, whereby weakness of the abdominal wall is prevented, and the figure speedily assisted to regain its natural shapeliness. The price in white coutille is 18s. 9d., or in the pure wool, natural colour, 23s. 6d. All sizes are kept in stock and can be fitted by a lady attendant in a private room. The "Hypogastric" is a belt specially designed for those with very large or pendulous abdomens. It is also highly recommended in cases of displacement of the uterus. A peculiarly-constructed flexible steel spring with a well-cushioned pad maintains an equable and firm pressure above the pubes. Then there are special belts for umbilical hernia and for floating kidney, both excellent in every way as regards construction, and fitted with proper pads for pressure, &c. The "Domen" abdominal belt bandages are a line deserving of special attention. They are easily adjustable, and take off that ugly ridge where the corset ends, which is sometimes so noticeable in those whose figures incline to corpulency. There is one particularly nice design for evening wear which does not add in any way to the bulk, and draws the hips into elegant shape. It is easily adjustable to the corset.

In conclusion I would draw attention to the superior excellence of the "Domen" pregnancy corset. It is made entirely of elastic material, which admits of easy expansion where most needed, at the same time affording uniform support and pressure. The elastic band in front gives support from below and can be drawn in or let out as required through the medium of buckles and laces. In white the price is 22s. 6d., or in natural colour, pure wool, 27s. 6d.

A USEFUL CASE-BOOK.

Messrs. Wodderspoon and Co. are to be congratulated on an excellent little case-book they have just brought out from the design of a nurse under the initials of S. G. It contains ample room for notes, and has a well-arranged column for the doctor's remarks opposite the nurse's report. It is simple in construction, which is a great point, especially to busy medical men, who will not be at the trouble to hunt for information in the more complicated forms one too often sees produced by private nurses. I wish this "clinical case-book" the success its merits deserve.

ACCESSORIES FOR THE TOILET.

AT 126 Oxford Street Madame Blanche Leigh has opened a branch of the establishment which she has carried on in the Rue de la Paix, Paris, for the past two years. Mme. Blanche Leigh makes a speciality of antiseptic toilet soaps, of which several kinds are to be found at 126 Oxford Street, ranging in price from 6d. to 3s. 6d. per cake. Here are also all sorts of dainty accessories to the toilet, sachets of powder for softening water, scents, scent sachets, &c. Mme. Leigh is also showing some charming Christmas gifts in the shape of purse bags, price from 15s. to £4; pincushions, 5s. to 35s.; and cases containing soap, scent and powder, price 16s. 6d. and upwards. Every article on sale here is distinguished for the dainty manner in which it is put up.

A PAPER TOY-MAKER.

MESSRS. SIMPKIN MARSHALL have published one of the nicest presents for little people that we have seen this Christmas. This is "The Young Paper Toy-maker," which contains in book form every manner of object which with the aid of a pair of scissors it is possible for little hands to convert into toys. There is dolly and her clothing, numerous animals, furniture, houses, Punch and Judy show, and so many other articles that it is impossible to enumerate them here. The models are drawn with broad lines on very stout paper. Each is accompanied by explicit and simple directions. Further, with the book a box of coloured crayons is issued, so that the toys can be appropriately coloured before cutting out. And all this for the small price of 1s. Fancy what hours of entertainment are provided by this useful and instructive little present.

### Appointments.

COTTAGE HOSPITAL, DAWLISH.—Miss Edith Daniel has been appointed matron. She was trained at the Royal Devon and Exeter Hospital, and has since been sister. She has also done a considerable amount of work on the private staff.

WORTLEY UNION WORKHOUSE INFIRMARY, GREENSIDE, SHEFFIELD.—Miss Mary Jarvis has been appointed superintendent nurse. She was trained at Sheffield Royal Infirmary, and has since been nurse at Hitchin Union Infirmary.

BROMLEY AND BECKENHAM JOINT HOSPITAL. — Miss F. H. Whitmarsh has been appointed matron. She was trained at the New Infirmary, Lewisham, and has since been charge nurse at the Bromley and Beckenham Joint Hospital. Miss Whitmarsh holds the L.O.S. certificate.

COTTAGE HOSPITAL, OTTERY ST. MARY, S. DEVON.—Miss Florence Gratwicke has been appointed matron. She was trained at the Children's Cottage Hospital, Cold Ash, Berks, and afterwards at the Royal Devon and Exeter Hospital, where she remained $5\frac{1}{2}$ years, and has since been private nursing for two years.

CARLISLE WORKHOUSE HOSPITAL.—Miss Mary Kervin has been appointed superintendent nurse. She was trained by the Nathan Workhouse Nursing Association for four years, and has since been nurse at Chatham Union Workhouse and charge nurse at West Hartlepool Workhouse Infirmary and Carlisle Workhouse Hospital.

GLASGOW WESTERN INFIRMARY.—Miss E. C. Shannon has been appointed matron. She was trained at Glasgow Western Infirmary for three years, and has since been charge nurse, and then constant matron, Stanley Hospital, Liverpool; matron, Grantham Hospital; superintendent of nurses, Scottish National Red Cross Hospital, South Africa; and night superintendent, Glasgow Western Infirmary.

MIRFIELD INDUSTRIAL INFIRMARY.—Miss Lucy Murray has been appointed nurse-matron. She was trained at Leeds General Infirmary. She has since been in temporary charge of wards in the Ida Convalescent Hospital; head nurse, Grantham Hospital; in charge of male medical and surgical wards, Bucks General Infirmary; night superintendent, Clayton Hospital, Wakefield; and home sister, Belfast Nurses' Home and Training School.

STIRLING DISTRICT ASYLUM, LARBERT.—Miss Jessie F. Mackintosh has been appointed night superintendent (assistant matron). She was trained at the Edinburgh City Hospital and the Ayr County Infirmary. She has since been attached to the Royal Scottish Nursing Institution, Queen Street, Edinburgh.

Miss Margaret Alison has been appointed assistant matron (day). She was trained at the Longmore Hospital for Incurables, Edinburgh, and the Western Infirmary, Glasgow.

Miss Frances Kippen has been appointed assistant matron (day). She was trained at the Longmore Hospital for Incurables, Edinburgh, and the West Hospital, Aberdeen; and has since been sister-in-charge at the Hospital for Incurables, Manchester, and attached to the private nursing staff of the Edinburgh Trained Nurses' Association.

### Minor Appointments.

KENSINGTON INFIRMARY.—Miss Ruth Annie Bedford and Miss Annie Mabel Harding have been appointed sisters. Miss Bedford was trained at Altrincham General Hospital, where she has since been nurse. She has also been charge nurse at Wakefield Infirmary. Miss Harding was trained at St. Saviour's Union Infirmary, and has since been staff nurse in that institution, charge nurse at Hanwell Ophthalmic School, and head nurse at Whitechapel Union Infirmary.

STANLEY HOSPITAL, LIVERPOOL.—Miss Christina Macintosh has been appointed staff nurse. She was trained at the Royal Southern Hospital, Liverpool, and has since been staff nurse at the London Fever Hospital.

BOLTON FEVER HOSPITAL.—Miss Frances McCarbie has been appointed charge nurse. She was trained at the Mater Misericordiæ Hospital, Dublin, and has since been engaged in district nursing at Liverpool and Bolton.

### Where to Go.

THE NEW GALLERY.—The Society of Portrait Painters are holding their exhibition at the New Gallery. The collection of works is a most interesting one, and the styles of portraiture are quite extraordinarily varied. Watts, Herkomer, Collier, Shannon, Hacker, Orchardson, and Ellis Roberts have contributed largely to the exhibition, which, therefore, has examples of the highest types of portrait painting, as well as those that are excellent and indifferent from the brushes of less well-known artists. One of the prettiest groups in the Gallery is by Mrs. Mary Waller, portraits of Joan and Enid, daughters of Lord George Campbell. The little maidens are in a background of apple-blossom, and are like two charming flowers themselves. It is quite an object-lesson to go from this study in pretty clear colour to one by Whistler called "The Lady of the Black Heart." In this room, which contains Whistler's work, there are other portraits which we think are carefully and wonderfully painted.

JANUARY 2.—Portman Rooms. Sir Squire Bancroft will read Dickens's "Christmas Carol," at 8.30., in aid of the Western Ophthalmic Hospital, Marylebone Road.

### Prize Distribution at Bristol General Hospital.

NO Gold Medal was awarded this year at the examination of nurses of the Bristol General Hospital, but the Silver Medal was awarded to Nurse C. C. Edwards, and Certificates of Merit to Nurse E. Bale and Nurse Hambleu. The following prizes were awarded:—

PHYSIOLOGY.—2nd year 1st prize, Nurse Page; 2nd year 2nd prize, Nurse Talbot; 1st year 1st prize, Nurse Frasberry; 1st year 2nd prize, Nurse E. Thomas.

ANATOMY.—2nd year 1st prize, Nurse Page; 2nd year 2nd prize, Nurse Talbot; 1st year 1st prize, Nurse Frasberry; 1st year 2nd prize, Nurse Gildford.

MEDICAL NURSING.—2nd year 1st prize, Nurse Page; 2nd year 2nd prize, Nurse Hadley; 1st year 1st prize, Nurse E. Thomas; 1st year 2nd prize, Nurse Frasberry.

The prizes were distributed by Mr. J. S. Fry, the Chairman.

### "The Hospital" Convalescent Fund.

THE hon. secretary begs to acknowledge with many thanks the receipt of 2s. 6d. from Miss G. M. E. Blackett, whose expression of good wishes for this fund is much appreciated

### Echoes from the Outside World.

### AN OPEN LETTER TO A HOSPITAL NURSE.

THOSE who had begun to fancy that the war in South Africa was really over, since Lord Roberts was on the sea, returning to his native land, must have been awakened rudely from their illusion by the news of the reverse which befell General Clements. The loss of a camp with a good deal of ammunition and 600 men taken prisoners, to which must be added the death of 5 officers and 9 men, and the disablement of 6 officers and 45 men, was not pleasant reading upon the anniversary of the battle of Colenso, and when the "saviour of the situation," as he was once called had resigned the reigns of government. But probably no one was less surprised than Lord Roberts himself. He knew, and stated openly, that such catastrophes were likely to occur for some little time yet, and Lord Kitchener may be trusted not to let the grass under his feet grow any longer than is necessary. On this particular occasion the Boers had attired themselves in khaki, and were clad exactly like the British, so that they were looked upon as friends till they began to fire. The fact of the large number of prisoners captured was much less serious than it at first appeared, because the Boers have now no facilities to feed or to retain our men, and consequently after stripping them of their accoutrements, are obliged to send them back after a few days. The same will probably happen with the 120 men of Brabant's Horse surrounded and captured near the Basuto border. Lord Methuen's report that he captured a whole Boer laager with 15,000 rounds of ammunition, 1,460 cattle, and 2,000 sheep did something to remove the uncomfortable impression made by the reverse on the Magaliesberg. Meanwhile, we hear of Lord Roberts at St. Helena, though looking well, with his arm in a sling from the injury he received when he fell off his horse. On his arrival in England he will go to Osborne for the night to see the Queen, and on January 3rd he will arrive in London and lunch with the Prince of Wales at Buckingham Palace. The proposed premature Thanksgiving Service at St. Paul's Cathedral has been wisely abandoned.

YOU will probably have noticed the reference a couple of weeks ago to the fact that during the Tsar's recent serious illness his only nurse had been his wife, and the fear expressed lest the Royal precedent should be followed by other wives with probably a less happy result. But according to a letter from Livadia, it seems that although wifely devotion was of course an important factor in causing the Tsarina to arrive at her determination to nurse her husband, another point which strongly influenced her was that by undertaking the charge of the invalid she would secure, what would be a great boon to both:—the chance of being alone and able to talk unrestrainedly in a way which, owing to their busy life, they find usually impossible. Strange to say, the strain seems to have told upon her much less than was expected. Her motherly anxiety was set at rest because every day the three little Princesses, who were sent to another house, came to see her, and she looked at them through the windows and thus satisfied herself that they were well and happy. They are spoken of as particularly sweet children, of whom the second, Tatjana, is the beauty, and they are always dressed in white. As they drive about, spending the afternoon very often with some other little girls, children of a general who lives close by, they chatter and laugh merrily enough, bowing with baby grace to those who greet them by removing their hats. Evidently for some time before the illness—which the Russians call Crimean typhus, a typhus specially prevalent in the Crimea, whilst the English papers term it enteric or typhoid fever—the Tsar was not in good health, for his appetite for the usual and most beneficial articles of diet had failed, and he lived largely on frosted oysters. It is recognised that oysters are never a very safe food, because they so easily become affected by impurities, but when frosted the risk is greater, because it is impossible to be certain of freshness, and the Tsar's cook is said to have been of opinion that the oysters were the probable cause of the illness. But it is hinted that the fever may be, as many another trouble has often been, a blessing in disguise, for the appetite which follows upon convalescence, and which the bulletins especially mention as being conspicuous in the present instance, should do much to build up the Tsar's constitution, and send him back to his work a stronger and a healthier man.

YOU will be interested to hear how the League of Mercy is getting on. The first annual meeting, held by the direction of the Prince of Wales, who, as you know, is the Grand President, was held on Friday in the fine Court-room at St. Thomas's Hospital, and among the presidents and lady presidents at the meeting were several peers, peeresses, and other persons of distinction. Not, of course, that the League is specially intended for persons of distinction. The humblest are as warmly welcomed to its ranks as the most exalted, and only the other day one of the lady presidents told me the pleasure she had experienced when a cook in the service of a friend expressed her desire to be enrolled as a member. Moreover, at the gathering to which I allude, a president declared that the League had met with great success in the poor district of Whitechapel, and that the working men had attended meetings and warmly co-operated with him. The progress made in twelve months is very satisfactory, sixty-five districts having contributed an average amount of £100 apiece. Of course the amounts were much larger in some districts than in others; but the shilling subscriptions tell up wonderfully, and it is really within the power of those who contribute the minimum sum to make the League a vast and far-reaching agency in the interests of the hospitals. It has already shown that it has come to stay, but you can safely tell your acquaintances when you ask them to join that it is as yet only in its infancy. I always think that it is a particular gratification to be associated with an organisation in its youth, and to help in its development.

LADIES who live in the country almost all the year round frequently complain that though it is easy enough to amuse themselves in the summer, if they do not hunt in the winter and the clerk of the weather refuses to supply ice for skating, amusement is limited to walking, driving, and occasional social gatherings. To these the Ladies' Rifle Club which has been recently started for Newbury and the surroundings by Mrs. Carstairs, of Welford Park, whose husband, Captain Carstairs, is serving in South Africa, will probably suggest a welcome and improving diversion, and the example is likely to be largely followed by others of the fair sex who live near a large county town or in a populous country district. The enthusiasts at Newbury are to be instructed in the use of the rifle by an ex-drill sergeant, and it would be rather amusing to know his private opinion of his new recruits. As a rule ladies are bad markswomen because so little is done to train their eyes; but with practice they would probably improve immensely, and even if they are never called upon to defend their country they may find the power of shooting straight useful enough some time or other. Lately, in Ashanti, China, and South Africa the woman who could, on emergency, handle a gun properly, must have felt far safer than her sister who had never pulled a trigger in her life.

### Mrs. Creighton on District Nursing.

BY OUR OWN CORRESPONDENT.

AN interesting function takes place each year in connection with the Hammersmith and Fulham District Nursing Association, when friends give in their collecting books, and Miss Curtis and the Nurses are "At Home." There was a good programme of music on Thursday, and a houseful of visitors; the nurses dispensed tea in their sitting-room, and Mrs. Creighton kindly came and delivered a short address. Having read the list of collectors for the year, with the amounts collected, Mrs. Creighton suggested that it would be interesting and useful to know the number of people—many of them poor—who had contributed to the Association.

"We know," she said, "the value of the widow's mite in the eyes of the Lord," and a small sum was just as valuable as a large one, since it showed the interest taken in the Association's work; it was not a question of large sums, but of rousing the interest of a large number of people. The money collected yearly in this way had gone up during the last ten years from £37 18s. 9d. to £106 10s. 9d.; and there had been a rise of over £40 when, about three years ago, the nurses had moved into their present house, Carnforth Lodge.

### NURSES AND THE HOUSING QUESTION.

Mrs. Creighton pointed out the close connection between the problem of overcrowding and district nursing. Much illness was simply the result of too early moving into a newly-built house; and doctors were well-acquainted with the symptoms of a damp house, damp bedding, ill-fitting windows, &c. There were roads in some parts of this district which were so deep in mud that one might, she thought, almost as well be on the veldt; the amount of sickness in a rapidly increasing neighbourhood, with its consequent evils, was not to be wondered at. It showed that the work of the district nurses needed constant development.

### THE NURSE'S WORK.

There were, she continued, moments in life when everyone realised the amount of sickness there is in the world; such moments came when sickness was in one's own family. One realised then that the comforts of life, and everything that money could give, were often unavailing, and at such times the sense of the pain of the world was almost unendurable. She thought these times were given in order that the sense of pity might be roused. Nurses did much to soothe suffering; but one most important part of their work was to prevent sickness by teaching people the laws of health.

### WORK FOR THE NURSES.

Everyone could not be a nurse; if it were so, there would be no patients, and we should be like the people in the Scilly Isles, who earned a precarious living by taking in one another's washing. Alluding again to the work of collecting for a nursing institution, Mrs. Creighton said it was distinctly a work of mercy; it should not be looked down upon. Indeed, we needed to raise our ideas about begging, which would, she thought, have a large part to play in the future; and it was especially a Christ-like work when done by the poor for the poor. She concluded with the hope that there would be a steady increase of interest in the work of the Association.

The Rev. Prebendary Snowdon having thanked Mrs. Creighton for her address, and alluded to the serious illness of the Bishop, Mrs. Creighton replied, and added that the doctors were now very pleased with their patient's condition, that indeed they had that day made a great many jokes, which was always a good sign. They were, however, anxious the diocese should know that the Bishop would not be able to resume his full duties for some long time yet. The programme of music and recitations was then continued. Among other guests were Miss Bulloch, head of the Battersea Nurses' Home, and several of the local clergy and doctors, with whom a function at Carnforth Lodge is very popular. The house is old and picturesque, and, with its large garden—in summer gay with hollyhocks—is a pretty bit of "vanishing London."

### For Reading to the Sick.

CHRISTMASTIDE.

"GOD was made manifest in the Flesh."

Love came down at Christmas,
Love all lovely, Love Divine;
Love was born at Christmas,
Star and angels gave the sign.

Worship we the Godhead,
Love Incarnate, Love Divine;
Worship we, our Jesus:
But wherewith for sacred sign?

Love shall be our token,
Love be yours, and love be mine,
Love to God, and all men,
Love for plea, and gift, and sign.

*C. Rossetti.*

Once more we are entering upon the blessed season of Christmas. Once again God in His infinite mercy has spared us to rejoice in the return of the day which 1,900 years ago saw the birth of the Holy Child at Bethlehem. We have waited for this sacred time, each in our different way as circumstances and occasion have permitted. Perhaps to many it has come not in every way as we had pictured it to ourselves, but in quite other. In the way which sooner or later comes to all whom God "calls apart" when He would speak to their souls, by suffering, bereavement, or disappointment. In the silence of the blessed night, as the Christmas bells clash out the good tidings of "Peace on earth, goodwill to men," it is hard, perhaps, for us to join in their happy message. Our hearts are filled with longing to join in the social joys from which we by sickness or sadness are deterred, or there is some root of bitterness springing up and poisoning the springs of charity and love in our hearts, some remembered injury harder to bear than physical suffering dealt by a hand we loved, and trusted. If this be so, God knows best! He has seen, He has known what is necessary for the perfecting of our character. In love He has stricken us, in love He will raise us up again. And as the joyous sounds break out "answering each other through the mist," let us lift our hearts for one moment in reverent adoration. In fancy let us picture the homely scene of the Gallilean shepherds keeping watch over their sleeping flocks by night. The night was dark, but dawn was near. It was very likely at that moment, the darkest of the night which immediately precedes daylight, that they were startled by the presence of the angel bringing the Divine message: "Fear not, for I bring you glad tidings of great joy which shall be to you and to all people." At last the promise had been fulfilled: the expected Saviour was born who was to bring salvation to the suffering world. "And suddenly there was with the angel a multitude of the heavenly host praising God, and saying, 'Glory to God in the highest, and on earth peace, good will toward men.'" Yes! we cannot give glory to God if we cannot from our hearts say also, "good will to men." We must ask Him to give us grace to say it sincerely, and then we can rejoice with those who at this time are gathered in happy family union, while we, visibly alone only, will join our prayers to those of the saints—

"Who love Thee, and are loved by Thee;
Until the day break, and till the shadows flee."

And then, if our Christmastide cannot be a Merry, it will be what is far better, a Blessed and a Peaceful one.—*F. B.*

Though from the glad home throng at Christmastide
Dear ones are gone for ever,
The far-off Land seems near where they abide,
While naught can mar or sever
The unity of praise. Christ's holy birth
Is hymned with joy on high—with peace on earth.

Therefore (to us such wondrous grace is given)
With high angelic throng,
And all the blessed company of Heaven,
We join in holy song.
No longer parted while we meet in praise,
Most sweetly near on these our Holy Days.

*C. M. Prevost.*

### Everybody's Opinion.

### A SUGGESTION WANTED.

"PEACEFUL" writes: A nurse-matron at an isolated cottage hospital would be so glad of any suggestion. She has learnt singing but has no musical instrument, and needs to ascertain the right note in learning anything new. A tuning-fork is not sufficient. She has to take the lead when the patients sing, but she is often without any patients, and when alone singing would help to brighten her life very much. She cannot afford anything expensive.

### RULES IN PRIVATE NURSING.

"NURSE ELSIE" writes: I always make it a rule to count the time from the moment the dose is taken, and I think this is the general rule among my fellow nurses. I note down upon my tablets the exact time the dose is given at once, so that there is no danger of forgetting. I am a nurse of several years' experience, and have done a great deal of private nursing; but I have never been asked to sleep in a convalescent man's room, and if the patient were really convalescent, and only needing attention once or twice during the night, he surely would not wish it. I think the matron "A Watcher" speaks of must be the exception to the general run of matrons, whom I have always found most particular in that respect, especially with regard to young nurses.

"JESMOND" writes: The letter signed "A Watcher" must have been written by a very young and a very indifferently trained nurse. She says: "Supposing the patient sleeps an hour or more beyond the time the (say 4-hourly) dose is due, is the time counted from the moment the dose is taken?" Surely this is a question which no one but the doctor in charge of the case is qualified to answer. I think if "A Watcher" does private work long she will find that each doctor she works for will expect her to consult him as to whether he will have the patient wakened or not for medicine, nourishment, &c., and his decision will depend entirely on the nature of the case and the general condition of the patient, and will probably not be the same in any two cases. Probably every private nurse will agree with me that it is much the wisest and safest plan to find out the wishes of the doctor in each individual case, and to carry out those wishes to the letter, thus throwing all responsibility of treatment on his shoulders, and not in any way laying herself open to the charge of usurping the doctor's place. "A Watcher's" second question as to the advisability of a private nurse sleeping in a convalescent man's room is, I think, one which only a very indifferently trained nurse would put. I was taught that patients in the convalescent stage require quite as much looking after as they do at the acute stage of their illness, especially if the illness has been at all severe. As to the age of the nurse, I entirely fail to see what possible difference that can make. Surely if a woman has gone through the regular three years' course of training in a general hospital (and no nurse is competent to undertake private work without doing so) she has learnt that patients, male or female, regard the nurse simply as a necessary, and in most cases a very welcome, adjunct to their illness. I am not one of those people who think that because a woman is a nurse she should lay aside all feelings of modesty, not to say common decency; but I do say that a woman who is capable of putting such a question as that of "Watcher" into words would do well to think seriously as to whether she has not mistaken her vocation in taking up such a profession as nursing sick people. In very many cases a nurse has only herself to blame if she fails to obtain the respect to which nurses, of all women, are surely most entitled from convalescent no less than from sick male patients.

### A PRIVATE NURSING INSTITUTION.

"NURSE" writes:—After a term of two years' training in a large Midland fever hospital, I applied to several general hospitals, with a view to entering as a probationer for training. At last I succeeded in having my application accepted, but was informed that it would probably be some months before a vacancy occurred. Meanwhile I applied to one of the many advertised private nursing institutions, until the time came that I could enter for my general training. In due course I was appointed to undertake medical and fever cases, also to do temporary duty in the local fever hospitals. On my arrival at the so-called institution I was not a little surprised to find a small house of six rooms, two of which were sitting-rooms, two bedrooms (each containing three camp beds, for the nurses, and two smaller bedrooms. I was shown the room in which I was to sleep, with instructions to start in the morning for my first case. I returned in a few weeks from my case to the "Home," and was sent to a fever hospital. On returning from the latter I was sent next day to a medical case. My thoughts were now on the infection, for I had been in a ward with patients suffering from scarlet fever. After this I made inquiries as to quarantine, and found that no precautions were taken, which by this time did not surprise me much. Some of the nurses returned to the "Home" from nursing smallpox, and spent their time of quarantine there. Of course they were supposed to keep to the room set apart for them for the time. But they went out each day for exercise, during which interval they mixed with the other nurses, having to both sleep and dine in the same room. This trained nurses' institute (as it is called) is managed by a private individual, whose only object is to make it, as it proves to be, a money-making concern. I would not say that all such "Homes" or "Institutions" are like it, but I give my own experience. To nurses about to enter a home or institution I would suggest the strictest inquiries before becoming one of the staff The forms received will probably look very plausible, but after all one cannot be too careful."

### CORPORAL PUNISHMENT IN INDUSTRIAL SCHOOLS.

"MR. LLEWELLYN W. WILLIAMS, B.Sc.," Hon. Sec., Society for the Reform of School Discipline, Park Terrace, Glasgow, S., writes: According to the report in your issue of November 17th, you seem to have received a rude shock at Miss Morten's statements with regard to the infliction of corporal punishment in some of the girls' industrial schools. I should like to mention that corporal punishment on boys and girls in the ordinary day schools is not so rare or so mild as those who desire an easy conscience would imagine. In the Glasgow School Board, a special officer is employed to attend to the numerous complaints by parents. In the Cathcart School Board I have myself taken an approximate census of the inflictions of corporal punishment on boys and girls, which shows that in many of the classes 20 per cent. of the pupils are thrashed each day. As recently reported by the *Church Gazette*, it is not uncommon to thrash children for obeying the Fifth Commandment. The *Church Gazette* explained this statement thus: "If a woman is in child labour and keeps her daughter at home to manage the necessary household duties, that same little mother's help on returning to school next day is thrashed because she has no written excuse." On the other hand, however, much progress has been made in recent years. Corporal punishment is entirely abolished in the schools of France, Holland, Belgium, Turkey, New York, Chicago, Cleveland, parts of South Australia, and many British schools, including Garnethill public school, Glasgow, Newtown School, Waterford, and Ackworth School, Pontefract, where natural and educative methods of discipline are substituted with most excellent results. At the beginning of this month corporal punishment was abolished in Griffin Infant School, Blackburn. Also, it is evidently the desire of the Education Department to step into line with other civilised communities, as is evinced by their recent instructions:—"Discipline maintained by harshness or by the aid of corporal punishment is not to be considered satisfactory."

### To Nurses.

WE invite contributions from any of our readers, and shall be glad to pay for "Notes on News from the Nursing World," or for articles describing nursing experiences, or dealing with any nursing question from an original point of view. The minimum payment for contributions is 5s., but we welcome interesting contributions of a column, or a page, in length. It may be added that notices of entertainments, presentations, and deaths are not paid for, but of course, we are always glad to receive them. All rejected manuscripts are returned in due course, and all payments for manuscripts used are made as early as possible at the beginning of each quarter.

### Notes and Queries.

**The Editor is always willing to answer in this column, without any fee, all reasonable questions, as soon as possible.**
**But the following rules must be carefully observed :—**
1. **Every communication must be accompanied by the name and address of the writer.**
2. **The question must always bear upon nursing, directly or indirectly.**

**If an answer is required by letter a fee of half-a-crown must be enclosed with the note containing the inquiry.**

*Home Hygiene.*

(117) Will you kindly tell me of a book, giving notes on nursing and home management, which would help a nurse to talk to mothers in her district ?—*Nurse B.*

"The Mother's Help and Guide to the Domestic Management of her Children," by P. Murray Braidwood, M.D. (Scientific Press.)

*Letters Forwarded.*

(118) Can you tell me of any very reliable place to which I could have my letters addressed, and whence they would be forwarded, without delay, on the payment of a small fee ?—*Nurse W.*

You might make arrangements with a local stationer.

*Dispenser.*

(119) Would you tell me if it is very expensive to be properly qualified as a dispenser? Also, if it takes a very clever person to pass the examina tions ?—*Dred.*

Both money and brains are required by those who would qualify as dispensers. An article by the Resident Dispenser at Ryde appeared in the HOSPITAL NURSING MIRROR of September 8th last, which should be useful to you.

*"Rest" Treatment.*

(120) Can you tell me what the "Rest" treatment is? Is there a small book dealing with the subject, as my patient may have to undergo it ?—*E. B.*

By the "Rest" treatment we presume you allude to that known as the "Weir Mitchell" treatment. If so, you will find chapters on the various new treatments in "Nursing : its Theory and Practice," by Lewis, price 3s. 6d.

*Crippled.*

(121) A domestic servant, crippled some years ago by the amputation of her leg, is very anxious to know of some means by which she could earn her living. She is 33 now, and, being absolutely friendless, is in the workhouse. She would be exceedingly grateful for advice.—*Sympathiser.*

So much depends upon the woman's own capacity in other ways. There are many trades which are independent of the power of walking—dress making, millinery, &c. Why not advertise in your local paper? Probably someone would have something to do whereby willing hands could earn a livelihood.

*Hot-air Treatment.*

(122) Can you tell me of any institution using the hot-air treatment for rheumatism with success? How could I get a patient there, either free, or by paying a small sum ?—*Sister Marie.*

You can obtain full particulars from the Matron of the Dowsing Institution, 28 York Place, Baker Street, W., or from Mr. Tallermann, Welbeck Street.

*Hot-water Dish.*

(123) Will anyone, who may know of a good form of hot-water dish or other appliance for carrying joint from kitchen to wards, kindly give me information as to where it may be obtained ?—*Lady Superintendent.*

Will a reader kindly recommend ?

*Midwifery.*

(124) Kindly give the names of two or three hospitals where a free or cheap training in monthly nursing and midwifery is given.—*Enquirer.*

There is no free training in nursing to be had. Pupils must always give an equivalent, either in service or in money. The British Lying-in Hospital, Endell Street; the City Lying-in Hospital, City Road; and the East End Mothers' Home, Commercial Road, E., all give cheap and efficient courses of training in maternity nursing.

*A Breach of Etiquette.*

(125) Six months ago I offered myself as candidate for probation at a London hospital. I was sent a form which I duly filled in, but of which I have since heard nothing. Would it be a breach of etiquette to write again, or to apply elsewhere ?—*Isobel M.*

You may apply to a dozen different institutions at the same time if you like. If a fortnight passes without your receiving a reply to your application, you may be sure that it has not been entertained.

*Infants' Feeds.*

(126) 1. Can anyone tell me the reason of the unpleasant smell when the rubber discs are removed from the sterillsing bottles of the Soxhlet apparatus? The milk tastes sweet and keeps good for two or three days. 2. What are the right quantities of pure milk with which to feed a baby brought up by hand ?—*Enquirer.*

1. Possibly it is the smell of the warm rubber that is unpleasant. If, however, the rubber smells "sour" it had better be sterilised by boiling. 2. You would find Dr. Pritchard's little work, "The Physiological Feeding of Infants" (price 1s.), just what you want.

*Insomnia.*

(127) Last winter I studied until midnight, and now I cannot sleep until after that hour. I am out all day and busy, yet when I go to bed at 10 p.m. I lie awake until nearly 1 a.m. Can anyone tell me of a simple remedy for resting well ?—*Agnes.*

You have to break yourself of a bad habit. Persevere in going to bed at 10 p.m.; at bed time put your feet into hot water, and after you get into bed, take a glass of hot water. Don't think of exciting topics.

*Domestic Occupation.*

(128) Will a person who has followed a domestic occupation be admitted as probationer to a hospital? I have heard that it is a barrier ?—*I. D.*

A few hospitals prefer probationers coming from the ranks of domestic service. The Middlesex Hospital, Mortimer Street, W., is one: for others, see the "Nursing Profession : How and Where to Train."

*Imbecile Youth.*

(129) I should be greatly obliged if you can tell me of an institution where an imbecile youth of 20 could be received as paying patient. One of the largest asylums in the northern counties refuses to admit him because he is past the age to benefit by education.—*Sah Tee.*

Possibly you would have better success at Earlswood Asylum for Idiots and Imbeciles, Redhill (office, 36 King William Street, London, E.C.), and there paying over 12s. at the Western Counties Idiot Asylum, Starcross, Exeter have special privileges. If unsuitable, there are others, for which see lists in "Burdett's Hospitals and Charities."

*M. R. B. N. A.*

(130) 1. Having had two years' general and two years' fever training, am I eligible as member of the Royal British Nurses' Association? If so, please give me the address. 2. Could I join the Army Nursing Reserve ?—*Rajah.*

1. The Royal British Nurses' Association, 17 Old Cavendish Street, Cavendish Square, W. 2. Apply the Hon. Secretary, Army Nursing Reserve 18 Victoria Street, S.W.

*Open-air Sanatoria.*

(131) Please tell me if there are any open-air sanatoria where patients are received free, or for a small charge ?—*Flo. T. B.*

Most of the consumption hospitals have adopted this treatment to some extent. Apply to the secretaries of the National Hospital for Consumption, Ventnor, and to the North London Hospital, Mount Vernon, Hampstead, N.W. The terms are from 10s. a week.

*Leprosy.*

(132) 1. Can you give me any information respecting the nursing of leprosy? 2. Are services given, or is a salary paid the nurse ?—*Lorna Doone.*

1. A nurse may perhaps encounter isolated cases of leprosy in the work of a skin hospital, not otherwise. 2. The services of a trained nurse in skin hospitals are usually paid for.

*Pelvis.*

(133) Will you kindly tell me if a pelvis can be hired for private lessons in midwifery? If so, where ?—*Nurse Sara.*

Apply to the Midwifes' Institute, 12 Buckingham Street, Strand, W.C., or to a local dealer in surgical and medical appliances.

*Certificate.*

(134) For two years I have been a nurse in an infirmary of 40 beds. have also a certificate for First Aid. I am now told that I cannot obtain a position other than probationer in a large hospital, as I am not a certificated nurse. Could I get a certificate by remaining another year here? —*Disappointed.*

Enter a good hospital as probationer and earn your three years' certificate from a recognised school. You will never be sorry; and you can choose one where the salary begins at once.

***Massage.***

(135) 1. "Novice" would be glad to learn where, in London, to be trained in massage? 2. Also if it is advantageous for a trained nurse to understand either, or both the Schott Nauheim and the Weir Mitchell treatments?

1. The National Hospital for the Paralysed and Epileptic, Queen's Square, Bloomsbury, W.C. 2. It is advantageous for a nurse to learn as much as possible in all branches of her profession.

*Dispensers.*

(136) Will you kindly tell me if there is much demand for lady dispensers with hospital training; and, if so, the average salary, and whether or no board is given ?—*Margaret E.*

Lady dispensers, if well qualified, have an opening field of enterprise before them. The salaries of assistant dispensers are from £40 to £60; those of dispensers are of course higher, with or without board.

*Nurses' Co-operation.*

(137) Is there a Nurses' Co-operation in Liverpool? Please give me the address.—*F. G. S.*

Of the more important nursing institutions in Liverpool none seem to be conducted on the co operative system.

*Mothers' Meetings.*

(138) Will the Editor kindly tell the Matron of a Convalescent Home of a book on such subjects as exercise, ventilation, cleanliness, food, cooking, dress, &c., suitable for reading at a mothers' meeting?

See reply to Nurse B., 117.

*Male Nurse.*

(139) 1. At what hospital are male nurses trained? 2. What salary is given during the period of training. 3. How is the Medico-Psycological certificate obtained.—*A. S.*

1. The National Hospital for the Paralysed and Epileptic, Queen's Square, Bloomsbury, W.C. 2. Salary first year, £12; second year, £20. 3. By three years' service in a lunatic asylum, where the attendants are trained for the Society's examinations. Lists of asylums providing such instruction are to be found in the "Nursing Profession : How and Where to Train."

*Postage.*

(140) 1. Can I obtain THE HOSPITAL in Paris? If so, where? 2. Are the postal authorities justified in calling one copy of THE HOSPITAL two papers, and demanding an extra penny on delivery?

1. You had better subscribe and have it sent from the office. 2. THE HOSPITAL NURSING MIRROR is a section of THE HOSPITAL, and the postage of which paper is, of course, a halfpenny.

**Standard Books of Reference.**

"The Nursing Profession : How and Where to Train." 2s. net; post free, 2s. 4d.
"The Nurses' Dictionary of Medical Terms." 2s.
"Burdett's Series of Nursing Text-Books." 1s. each.
"A Handbook for Nurses." (Illustrated.) 5s.
"Nursing : Its Theory and Practice." New Edition. 3s. 6d.
"Helps in Sickness and to Health." Fifteenth Thousand.
"The Physiological Feeding of Infants." 1s.
"The Physiological Nursery Chart." 1s.; post free 1s. 3d.
"Hospital Expenditure : The Commissariat." 2s. 6d.
All these are published by THE SCIENTIFIC PRESS, Ltd., and may be obtained through any bookseller or direct from the publishers, 28 and 29 Southampton Street, London, W.C.

